# Origins of Aminergic Regulation of Behavior in Complex Insect Social Systems

**DOI:** 10.3389/fnsys.2017.00074

**Published:** 2017-10-10

**Authors:** J. Frances Kamhi, Sara Arganda, Corrie S. Moreau, James F. A. Traniello

**Affiliations:** ^1^Department of Biological Sciences, Macquarie University, Sydney, NSW, Australia; ^2^Department of Biology, Boston University, Boston, MA, United States; ^3^Centre de Recherches sur la Cognition Animale, Centre de Biologie Intégrative, Université de Toulouse, CNRS, UPS, Toulouse, France; ^4^Department of Science and Education, Field Museum of Natural History, Chicago, IL, United States; ^5^Graduate Program for Neuroscience, Boston University, Boston, MA, United States

**Keywords:** neuromodulation, biogenic amines, eusocial, social brain evolution, collective intelligence

## Abstract

Neuromodulators are conserved across insect taxa, but how biogenic amines and their receptors in ancestral solitary forms have been co-opted to control behaviors in derived socially complex species is largely unknown. Here we explore patterns associated with the functions of octopamine (OA), serotonin (5-HT) and dopamine (DA) in solitary ancestral insects and their derived functions in eusocial ants, bees, wasps and termites. Synthesizing current findings that reveal potential ancestral roles of monoamines in insects, we identify physiological processes and conserved behaviors under aminergic control, consider how biogenic amines may have evolved to modulate complex social behavior, and present focal research areas that warrant further study.

## Introduction

**Figure 1:**
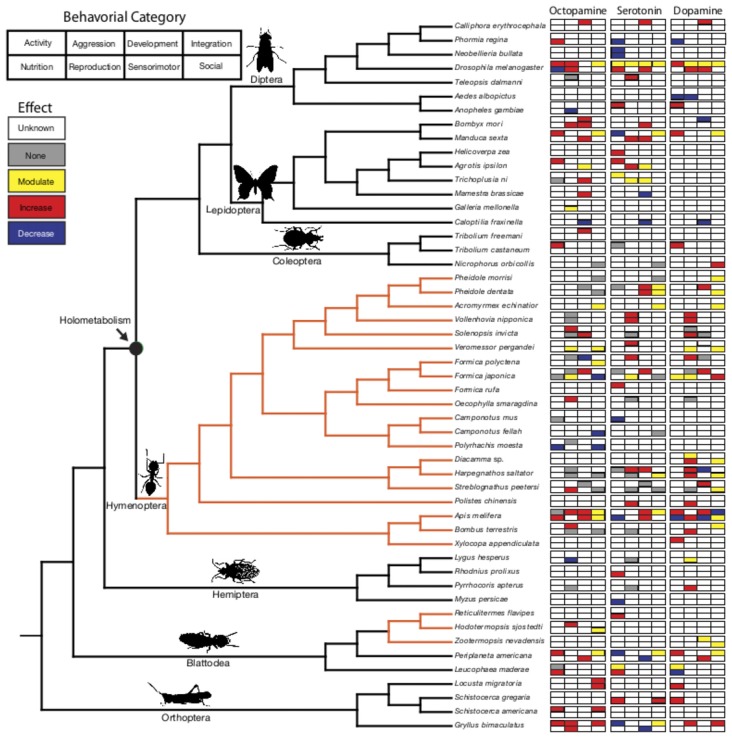
Phylogenetic relationship of biogenic amine function across the insects. Behaviors are organized into eight categories (activity, aggression, development, sensory integration, nutrition, reproduction, sensory motor, social function). The overarching trend of the behavioral effects for octopamine (OA), serotonin (5-HT) and dopamine (DA) in each of these categories is represented in the corresponding boxes. Within the phylogenetic tree, black lines indicate solitary/presocial species and orange lines indicate the evolution of eusociality. Insect images are from PhyloPic. http://phylopic.org

The ubiquitous biogenic amines octopamine (OA), serotonin (5-HT) and dopamine (DA) activate neural circuitry to regulate behavior (Libersat and Pflueger, 2004; Bergan, 2015). The phylogenetic distribution of these neuromodulators suggests a deep evolutionary history predating the origin of the nervous system (Gallo et al., 2016). With few structural modifications, monoamines are functionally diverse in insects (Roeder, 1999; Mustard et al., 2005; Blenau and Thamm, 2011). Conserved aminergic circuits (Kravitz and Huber, 2003; Barron et al., 2010; Perry et al., 2016) and patterns of receptor expression (Roeder, 1999; Blenau and Thamm, 2011) control behavior in diverse species across insect orders. However, how monoamine neurotransmitter systems served as preadaptations for the evolution of derived behaviors associated with the transition from solitary life to sociality in insects is poorly understood. Insect colonies show remarkable variation in structure and degree of integration of worker actions that could underscore complex social behavior. Using well-resolved insect molecular phylogenies (Wiegmann et al., 2011; Song et al., 2012, 2015; Moreau and Bell, 2013; Regier et al., 2013; Schmidt, 2013; Misof et al., 2014; Wang et al., 2014), we explore the evolution of neuromodulation of social behavior (Supplementary Table S1) by analyzing patterns of monoamine function in solitary and social taxa (Figure 1; Supplementary Table S2).

## Social Decision-Making Systems and Behavioral Diversity in Insects

Two neural circuits regulate vertebrate decision-making: the social behavior network, controlled by neuropeptides and gonadal steroids, and the mesolimbic reward system, activated primarily by DA (O’Connell and Hofmann, 2011a,b). These circuits act in concert to regulate social interactions and evaluate stimulus valence, respectively, forming the social decision-making network (O’Connell and Hofmann, 2011b, 2012). Insect social decision-making systems are poorly understood in comparison, although behavioral influences of neuromodulators are well known (Supplementary Table S2).

Neurochemical and neuroendocrine analyses of complex social behavior in insects have largely been limited to the species-rich Hymenoptera (>150,000 species), which includes ants, bees and wasps with solitary, presocial and eusocial life histories. Solitary species are composed of individuals that live and forage alone and interact with conspecifics primarily during mating or territorial disputes. Presocial describes life histories that are intermediate between solitary and eusocial (Eickwort, 1981). Eusociality is defined by: (1) reproductive division of labor (the differentiation of fertile [queens and males] and sterile [workers] castes); (2) allomaternal care (cooperative care of immatures by workers); and (3) overlapping generations of reproductive and worker castes (queen longevity allowing coexistence with offspring). Varying degrees of sociality are found in a number of clades. Phase transitions from solitary to gregarious behavior occur in desert locusts (Order Orthoptera; Anstey et al., 2009; Ott and Rogers, 2010), beetles (Order Coleoptera) show multiple occurrences of the evolution of familial sociality, including biparental care (Costa, 2006; Cunningham et al., 2015; Panaitof et al., 2016), and one species of weevil is eusocial (Kent and Simpson, 1992). Solitary life histories predated eusociality in both the Hymenoptera (Wilson, 1971) and Isoptera, which diverged from cockroaches into entirely eusocial forms (Bourguignon et al., 2015). The evolution of a reproductive caste occurred once in ants and multiply in bees and wasps; diversification of workers, particularly in ants, has many independent origins (Oster and Wilson, 1978; Trible and Kronauer, 2017). Eusociality independently evolved in the Order Isoptera (termites, >3000 species; Thorne and Traniello, 2003).

Parental behavior, reproductive competition and foraging and defense strategies in solitary (Field et al., 2006, 2015; Thompson et al., 2014) and eusocial (Tibbetts, 2013) hymenopteran species reflect social decision-making, although neurochemical and neuroanatomical correlates of such systems are poorly understood (Ilies et al., 2015). For example, neural mechanisms underscoring vertebrate-like cognitive abilities, such as individual facial feature recognition in some eusocial wasps, are not known (Gronenberg et al., 2008; Sheehan and Tibbetts, 2011). Decision-making at the colony level is seen in collective (swarm) intelligence (Seeley, 2010; Jeanson et al., 2012; Sasaki and Pratt, 2012; Reid et al., 2015) and in part concerns worker interactions (Greene and Gordon, 2003; Greene et al., 2013) that may be causally related to brain neurotransmitter levels (Muscedere et al., 2012; Kamhi and Traniello, 2013; Kamhi et al., 2015; Hoover et al., 2016). Studies have focused on the aminergic control of worker interactions that contribute to social organization, including responsiveness to social signals and cues that regulate alloparental care, food exchange, nest construction, defensive behavior and foraging (reviewed in Kamhi and Traniello, 2013; Simpson and Stevenson, 2015; Hamilton et al., 2017). Studies have begun to explore genetic and epigenetic underpinnings of task performance and plasticity through state changes in behavior (Lucas and Sokolowski, 2009; Simola et al., 2016) that may involve neuromodulators.

## Behavior and Biogenic Amine Functions in Insects

Genes controlling behavior in solitary insects regulate social behavior in eusocial species (LeBoeuf et al., 2013) and affect sensory receptor evolution (Baldwin et al., 2014). Monoamine functions in solitary insects likely reflect this conservation, and appear to have been preadaptive for eusocialty. To understand the evolution of neuromodulatory systems in insects, we organized available data on aminergic control into eight behavioral categories: activity, aggression, development, higher-order sensory integration, nutrition, reproduction, sensorimotor functions and social functions (defined in Supplementary Table S1). Behaviors may span multiple categories, such as parental care and mate selection involving reproduction and derived social functions. Statistical tests showed similar patterns of monoamine function in solitary and eusocial species (Supplementary Figure S1), although small sample sizes constrain inferences. While data on biogenic amine regulation is variable and fragmentary, some patterns emerge suggesting that aminergic circuitry has shifted in function during the transition from solitary to social life. Monoamines have been co-opted for social functions through receptor and circuitry evolution and have gained novel functions to regulate social behaviors. For example, 5-HT (Alekseyenko et al., 2010, 2014; Bubak et al., 2014) and OA (Stevenson et al., 2000; Hoyer et al., 2008; Zhou et al., 2008; Stevenson and Rillich, 2017) increase aggression in solitary insects. In social insects, aggression is associated with the ability to pheromonally distinguish nestmates from non-nestmates (Stroeymeyt et al., 2010; Sturgis and Gordon, 2012), and OA is implicated in improved nestmate recognition (Robinson et al., 1999; Vander Meer et al., 2008; Kamhi et al., 2015). OA may thus enhance sensitivity to pheromonal cues and regulate social interactions similarly in both solitary and social insects.

DA, 5-HT and OA are involved in regulating metamorphosis in solitary insects (Nässel and Laxmyr, 1983; Hirashima et al., 1999). In social insects, monoamines are associated with age-related behavioral changes and collateral physiological and neural development (Schulz et al., 2002; Seid and Traniello, 2005; Cuvillier-Hot and Lenoir, 2006; Wnuk et al., 2010; Giraldo et al., 2016). OA increases with age and is causally related to the transition from nursing to foraging in honey bees (Schulz et al., 2002). In ants, 5-HT, DA (Seid and Traniello, 2005; Cuvillier-Hot and Lenoir, 2006), and OA (Wnuk et al., 2010) increase with age; 5-HT, similar to OA in bees, is correlated with age-related initiation of foraging (Seid and Traniello, 2005) and sensitivity to pheromonal signals underscoring trail communication (Muscedere et al., 2012).

In respect to other behaviors, suppressing DA neurons in *Drosophila melanogaster* consistently inhibits aversive but not appetitive learning, whereas manipulating OA action produces the opposite pattern (Schwaerzel et al., 2003; Claridge-Chang et al., 2009; Aso et al., 2010). Similar patterns have been found in honey bees (Mercer and Menzel, 1982; Hammer and Menzel, 1998). However, appetitive learning in social insects must be considered in respect to the social context, where foraging is dependent on the nutritional state of the colony rather than the individual (Traniello, 1977; Seeley, 1989). OA increases the likelihood of successful foragers waggle dancing, which communicates information about food location and quality to nestmates; this demonstrates that an amine may be adapted to serve a colony-level function in food collection rather than benefit individual nutrition (Barron et al., 2007).

Biogenic amines appear to have gained new functions associated with the regulation of social organization. DA correlates with increased receptivity and mating in solitary insects (Pastor et al., 1991; Neckameyer, 1998; Chvalova et al., 2014; Brent et al., 2016), and reproductive state in many hymenopterans (e.g., Sasaki et al., 2007). Honey bee and some ant workers are reproductively capable; however, both ant and honey bee queens release a pheromone, queen mandibular pheromone (QMP), that inhibits worker reproduction (Fletcher and Blum, 1981; Hoover et al., 2003) by acting through DA circuitry (Harris and Woodring, 1995; Boulay et al., 2001; Beggs et al., 2007). Aggressive interactions between workers to control reproductive dominance also affect DA levels (Shimoji et al., 2017). These studies suggest that in both solitary and eusocial insects DA regulates reproductive state, and DA additionally may be integral to the maintenance of reproductive division of labor and the resolution of reproductive competition in eusocial species.

## Focal Questions

We identify four research areas, among several others, that are significant in the study of the neuromodulation of complex eusocial behavior.

### Altruism, Genes and Neuromodulators

Altruism is evident in the sterility of workers and their fatal self-sacrificing behavior. Developmental programming controls ovarian function, feeding the queen and alloparental care, and likely regulates defensive responses that decrease the survival of altruistic workers. Correlations among DA, OA, their receptors, ovarian development and honey bee worker responsiveness to social signals of fertility have been identified (reviewed in Simpson and Stevenson, 2015; Hamilton et al., 2017). As discussed above, worker fertility is controlled by QMP, which also causes workers to feed and groom the queen and activates brain genes associated with alloparenting (Grozinger et al., 2003). Workers showing higher ovarian activity are less likely to show queen-directed behaviors (Galbraith et al., 2015). Honey bee ovarian development is associated with the expression of a tyramine receptor gene (Thompson et al., 2007) and brain levels of the OA receptor Oa1 (Cardoen et al., 2011; Galbraith et al., 2015; Sobotka et al., 2016). QMP also modulates DA receptor gene expression, decreases brain DA levels, and reduces activity possibly by inhibiting DA function in young workers (Beggs et al., 2007). Homologous systems appear to control reproduction in ants: QMP inhibits reproduction and DA may increase fertility (Boulay et al., 2001; Penick et al., 2014; Okada et al., 2015).

Together, these studies suggest that in eusocial insects DA regulates reproductive state and related social behaviors, which are key to altruism. Thompson et al. (2013) noted that “genes underlying altruism should coevolve with, or depend on, genes for kin recognition”; such genes specify recipients of altruistic actions. The regulation of polygyny (multiple queens) in ants and the direction of lethal aggression toward queens of a certain genotype, is under the control of the *Gp-9* gene, which codes for an odorant-binding protein (Gotzek and Ross, 2007). This indicates that chemical communication underscores strategies associated with inclusive fitness. Nestmate recognition may be causally related to monoamine levels (Kamhi and Traniello, 2013; Kamhi et al., 2015; Hoover et al., 2016) and altruistic defense. Self-sacrifice is associated with defensive specializations of “soldiers,” and may concern serotonergic circuits (Giraldo et al., 2013). Soldiers are more tolerant of risk; elevated monoamine levels or subcaste-specific receptor profiles may underscore their self-sacrificing behavior.

### Orchestration of Individual and Colony-Level Behavior

Social decision-making networks in vertebrates and eusocial insects function in different contexts and favor, respectively, individual reproduction and inclusive fitness. Concepts such as social brain theory (Dunbar, 1998), developed for vertebrates, may vary in its applicability to eusocial insects (Lihoreau et al., 2012). Similarly, neuromodulators play a key role in the “orchestration of behavior” (Sombati and Hoyle, 1984; Hoyle, 1985), but analyses of organizational mechanisms should distinguish between the regulation of individual behavior by monoamines and the control of emergent colony properties by pheromones to determine whether the orchestration hypothesis can explain the control of these two systems (Kamhi and Traniello, 2013). The circuitry of social networks underscoring division of labor and collective action may concern interactions of communicating workers, which have been considered to be functionally similar to neurons (Couzin, 2009; Feinerman and Korman, 2017). Similarly, pheromones are behavioral releasers that may parallel neurotransmitter functions in circuits. The role of the “colony brain” in emergent group behavior is therefore in part constructed from the neurochemistry of individual worker brains that modulate responsiveness to social cues and signals as well as social interactions and pheromonal communication systems that modulate group decision-making. Kamhi and Traniello (2013) hypothesized that worker interactions may cause neuromodulatory and behavioral synchronization in collective action, and that monoamine titers could regulate cyclical activity. Control processes analogous to neural synchronization in vertebrate brains may underscore colony-level behavior.

An emergent action that holds promise for such an analysis is cooperative foraging, a goal-oriented system in which chemical signals control colony behavior (Czaczkes et al., 2015). Foraging effort is modified by the responses of individual workers to pheromones that induce and terminate foraging activity by affecting individual and group decisions. The ability of workers to render decisions that modify colony-level responses may be related to worker physical caste or age. OA underscores subcaste-specific behavior in ants (Kamhi et al., 2015), and 5-HT in ants (Seid and Traniello, 2005; Seid et al., 2008) and OA in honey bees (Schulz et al., 2002) modulate age-related task transitions that involve striking shifts in stimulus environments within and outside of the nest. Biogenic amines may thus influence division of labor and collective action through changes in olfactory responsiveness.

### Nutrition and Biogenic Amines

Nutrition has diverse effects on social behavior, from group aggregation to brain physiology (Simpson and Raubenheimer, 2012; Lihoreau et al., 2015). Diet influences levels of brain monoamines, which are derived from amino acids such as tryptophan and tyrosine (Crockett et al., 2009; Wada-Katsumata et al., 2011; Fernstrom, 2013). In insects, 5-HT, DA and OA modulate feeding behavior (Braun and Bicker, 1992; Falibene et al., 2012) through regulatory mechanisms that may be conserved between solitary and social species (Dacks et al., 2003; Haselton et al., 2009; Neckameyer, 2010). Serotonergic fibers innervate the insect digestive system in species-specific patterns of distribution (e.g., Klemm et al., 1986; Molaei and Lange, 2003; Falibene et al., 2012; French et al., 2014). In eusocial insects, food is exchanged among colony members through trophallaxis. In the foregut, the proventriculus controls the transfer of food to the midgut (for individual worker metabolism) and its retention in the crop (to be shared with colony members). In solitary insects, 5-HT increases crop contractions (Liscia et al., 2012), enabling regurgitation (Stoffolano et al., 2008). In honey bees (French et al., 2014) and some ants (Falibene et al., 2012), serotonergic fibers innervate both organs; in honey bees, 5-HT antagonists affect crop and proventriculus contractions (French et al., 2014). In eusocial insects, 5-HT may thus have been co-opted for food sharing, reducing individual feeding behavior and enabling trophallaxis when the crop is full.

In ants, nutrient requirements differ among colony members: workers mainly feed on carbohydrates for energy, whereas larvae require protein for development. Colonies with larvae collect food with higher protein content (Abril et al., 2007; Dussutour and Simpson, 2008, 2009); communication of nutritional needs (Farina and Grüter, 2009; LeBoeuf et al., 2016) may thus modify food choices of foragers. Adjusting protein and carbohydrate intake in ants may affect nestmate recognition (Liang and Silverman, 2000; Buczkowski et al., 2005), social immunity (Kay et al., 2014), and colony behavior (Kay et al., 2010, 2012). However, we do not know how nutritional interactions affect forager monoamine levels and behavior. 5-HT underlies a dietary switch toward foods with higher protein content in fruit flies (Vargas et al., 2010), and OA and DA levels influence individual and social control of feeding in some ants (Wada-Katsumata et al., 2011). Nutritional ecology varies across social insect clades and may significantly impact monoamine levels and trophic behavior.

### Ligand and Receptor Coevolution

Biogenic amine receptor distribution in insect brains has been characterized primarily in fruit flies and honey bees (Blenau et al., 1998; Monastirioti, 1999; Blenau and Thamm, 2011; Sinakevitch et al., 2011). Receptor duplication has occurred throughout evolution and the same small number of monoamines appear to have been co-opted for use as ligands for duplicated receptors (Hauser et al., 2006). There are typically several types of receptors for each monoamine, which may lead to different regulatory mechanisms. For example, knocking out the 5-HT receptor d5-HT1A influences sleep in fruit flies (Yuan et al., 2006), whereas overexpression of receptor d5-HT1B reduces the ability to phase-shift in response to light cues (Yuan et al., 2005).

Receptor duplication and adaptation appears to have evolved before the divergence of fruit flies and honey bees, suggesting that solitary and social insects share common monoamine receptors (Hauser et al., 2006; Bauknecht and Jékely, 2017). If ligands, receptors, and downstream regulatory mechanisms are highly conserved across species, how have biogenic amine circuits evolved to control derived social behaviors? Monoamines may have species-specific effects on neural circuits, giving rise to different downstream regulatory effects and thus variable roles in modulating behavior. Activation of the DA receptor DopR1 increased stress-induced hyperactivity and modulated circadian-dependent activity through different neural circuits in fruit flies (Lebestky et al., 2009). Social insects may have evolved distinct neural circuits to regulate social behaviors using the same signaling molecules as solitary species. Exploring biogenic amine receptors and downstream regulatory pathways involved in insect behavior and derived social functions will advance our understanding of how the eusocial insect brain evolved perceptual and cognitive capacities in association with sociality.

## Conclusion

Broader sampling is required to gain phylogenetic insight into the evolution of aminergic control systems. Determining patterns of conservation and/or diversification of aminergic regulatory mechanisms of social behavior will benefit from studies of insect genera that include solitary and eusocial species. Despite the widespread activity of biogenic amines, functional patterns appear. 5-HT may control energy expenditure through feeding behavior and circadian rhythms, DA regulates fertility, thus modulating task performance in eusocial species, and OA modulates appetitive learning associated with feeding and nestmate recognition. Advances in epigenetics (Libbrecht et al., 2016), neurogenetics (Friedman and Gordon, 2016), and the integration of sociobiology and neurochemistry (Kamhi and Traniello, 2013) will aid in future research.

## Author Contributions

JFK, SA and JFAT compiled literature. SA performed statistical analyses and created associated figures, and CSM created the phylogenetic presentation of aminergic control of behavior. JFK, SA and JFAT prepared drafts of the manuscript. All authors contributed to the conception of the perspective, analysis and synthesis of material, manuscript revision and gave final approval for publication.

## Conflict of Interest Statement

The authors declare that the research was conducted in the absence of any commercial or financial relationships that could be construed as a potential conflict of interest. The reviewer VM declared a past collaboration with one of the authors CSM to the handling Editor.
