# Supplementary material for: Origins of Aminergic Regulation of Behavior in Complex Insect Social Systems
Source: Front Syst Neurosci. 2017 Oct 10;11:74. doi: 10.3389/fnsys.2017.00074 (PMC5641352; doi:10.3389/fnsys.2017.00074)
Supplement: Supplementary file 1 [file Table_1.pdf]

Supplementary Material

Origins of aminergic regulation of behavior in complex insect social systems

J. Frances Kamhi, Sara Arganda, Corrie S. Moreau, and James F. A. Traniello

***Correspondence:** Corresponding author: Franne.Kamhi@mq.edu.au

Data analysis and statistical methods

We compared the neuromodulation of biogenic amines (octopamine, OA; serotonin, 5-HT; and dopamine, DA) in solitary/presocial and eusocial insects using published data. Solitary and presocial species were combined in analyses because of the similarities in behaviors recorded. We created eight behavioral categories (activity, aggression, development, higher-order sensory integration, nutrition, reproduction, sensorimotor, and social functions; defined in Table S1) and five possible biogenic amine-behavioral category correlations (increase, decrease, modulation, none, and unknown).

For each species, we analyzed available published results and determined the type of biogenic amine-behavioral category correlation. We assigned a single value per species, biogenic amine, and functional category. *Increase* was assigned when increasing values of a given biogenic amine correlated with increasing values of a given behavioral category. *Decrease* was assigned when decreasing values of a given biogenic amine correlated with increasing values of a given behavioral category. *Modulation* was assigned when different doses correlated with different effects (for example, low levels of 5-HT in inside nest workers and high levels of 5-HT in outside nest workers) or if results in the literature were inconsistent. *None* was assigned when no significant relationship between the biogenic amine and the behavior was found. Empty cells in our figures indicate no information was available regarding biogenic amine function in a behavioral category for that species.

To represent evolutionary relationships among insects, we followed the phylogenies: Blattodea (Djernaes, et al., 2011, Coleoptera Misof, et al., 2014, Diptera Wiegmann, et al., 2011), Hemiptera (Song, et al., 2012, Wang, et al., 2014), Hymenoptera and Formicidae (Tree of Life: tol.org; Moreau and Bell, 2013, Schmidt, 2013), Lepidoptera (Regier, et al., 2013), Orthoptera (Song, et al., 2015); and (Misof, et al., 2014) for relationships among insect orders. The composite phylogenetic tree was constructed in Mesquite (Maddison & Maddison 2017). Images of insects were used with permission from PhyloPic (http://phylopic.org).

To compare effects of biogenic amines between solitary/presocial and eusocial insects, we built contingency tables for each biogenic amine and behavioral/functional category. We used the

Fisher exact test to determine whether social complexity (solitary/presocial versus eusocial) affected the function of biogenic amines.

Behavioral category	All insects	Solitary insects	Eusocial insects
**Activity**	Locomotion, escape response, circadian rhythms	n/a	n/a
**Aggression**	Defensive or agonistic actions, predation	n/a	Nestmate recognition
**Development**	Age-related physiological change	n/a	Age-related division of labor
**Higher-order integration**	Learning/memory, decision-making, emotion-like states, attention	n/a	n/a
**Nutrition**	Feeding behaviors, metabolism, sucrose responsiveness	n/a	Trophallaxis
**Reproduction**	Reproductive state, sex pheromone perception, female receptivity, oviposition	Parental care, mate selection	Colony foundation, reproductive division of labor
**Sensorimotor functions**	Sensory processing, reception, and response thresholds	n/a	n/a
**Social functions**	n/a	Parental care, mate selection	Trophallaxis, division of labor, collective action, brood care

Table S1: Behaviors comprising categories used for comparisons of solitary/presocial and eusocial insect aminergic control systems. n/a indicates no additional behaviors recorded in a given category.

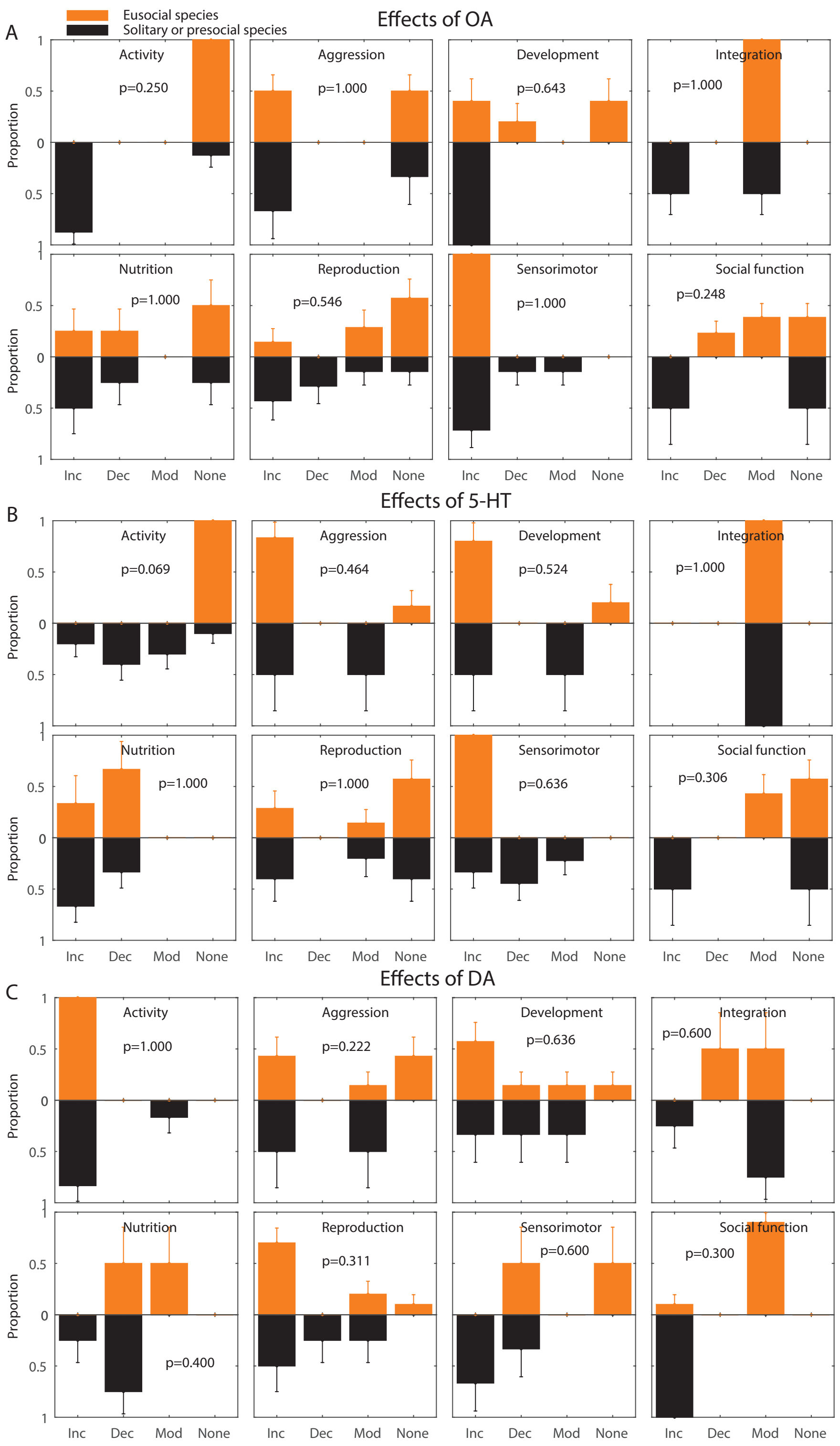

Figure S1: Distribution of effects of the biogenic amines in solitary/presocial and eusocial insects by behavioral category. **A.** Octopamine (OA); **B.** Serotonin (5-HT); **C.** Dopamine (DA).

Histogram coding: Orange (eusocial species); black (solitary and presocial species) Inc: increase; Dec: decrease; Mod: modulation; None: no effect. p-values calculated using a Fisher test on absolute numbers.

OCTOPAMINE								
Species	**Activity**	**Aggression**	**Development**	**Integration**	**Nutrition**	**Reproduction**	**Sensorimotor function**	**Social function**
Hodotermopsis sjostedti		Ishikawa, et al., 2016						Ishikawa, et al., 2016
Leucophaea maderae	Page, 1987				Cohen, et al., 2002			
Periplaneta americana	Goldstein and Camhi, 1991			Goldstein and Camhi, 1991			Goldstein and Camhi, 1991	
Reticulitermes flavipes								
Zootermopsis nevadensis								
Nicrophorus orbicollis								Panaitof, et al., 2016
Tribolium castaneum	Nishi, et al., 2010							
Tribolium freemani			Hirashima, et al., 1999					
Aedesal bopictus								
Anopheles gambiae						Fuchs, et al., 2014		
Calliphora erythrocephala			Nässel and Laxmyr, 1983					
Drosophila melanogaster	Sadaf, et al., 2015, Saraswati, et al., 2004, Yellman, et al., 1997	Hoyer, et al., 2008, Zhou, et al., 2008		Burke, et al., 2012, Müller, 1997, Schwaerzel, et al., 2003	Luo, et al., 2014	Certel, et al., 2010, Lee, et al., 2003		
Neobellieria bullata								
Phormia regina					Long and Murdock, 1983			
Teleopsis dalmanni		Bubak, et al., 2014						
Lygus hesperus						Brent, et al., 2016		
Pyrrhocoris apterus						Chvalova, et al., 2014		
Rhodnius prolixus								
Myzus persicae								
Acromyrmex echinatior								Smith, et al., 2013
Apis mellifera	Burrell and Smith, 1995	Burrell and Smith, 1995, Robinson, et al., 1999	Harris and Woodring, 1995, Schulz, et al., 2002a, Schulz and	Barron, et al., 2007, Burrell and Smith, 1995, Chen, et al., 2008, Hammer and	Braun and Bicker, 1992, Scheiner, et al., 2002		Kloppenburg and Erber, 1995, Mercer and Menzel, 1982, Robinson, et al., 1999,	Barron, et al., 2007, Schulz, et al., 2002a, Schulz and Robinson, 2001

			Robinson, 2001, Schulz, et al., 2002b	Menzel, 1995, Menzel, et al., 1999, Mercer and Menzel, 1982			Scheiner, et al., 2002	
Bombus terrestris		Bloch, et al., 2000				Bloch, et al., 2000		Bloch, et al., 2000
Camponotus fellah								Boulay, et al., 2000
Camponotus mus								
Diacamma sp.								
Formica japonica		Aonuma and Watanabe, 2012b	Aonuma and Watanabe, 2012a		Wada-Katsumata, et al., 2011	Aonuma and Watanabe, 2012a		Wada-Katsumata et al., 2011)
Formica polyctena		Szczuka, et al., 2013	Wnuk, et al., 2010					Wnuk, et al., 2010
Formica rufa								
Harpegthos saltator		Penick, et al., 2014				Penick, et al., 2014		Penick, et al., 2014
Pheidole dentata			Seid and Traniello, 2005					Seid and Traniello, 2005, Sandridge et al., unpublished
Pheidole morrisi								(Sandridge et al., unpublished)
Polistes chinensis								
Oecophylla smaragdina		Kamhi, et al., 2015						Kamhi, et al., 2015
Polyrhachis moesta		Koyama, et al., 2015			Koyama, et al., 2015			Koyama, et al., 2015
Solenopsis invicta		Vander Meer, et al., 2008				Boulay, et al., 2001	Vander Meer, et al., 2008	
Streblogthus peetersi			Cuvillier-Hot and Lenoir, 2006			Cuvillier-Hot and Lenoir, 2006		Cuvillier-Hot and Lenoir, 2006
Veromessor pergandei						Muscedere, et al., 2015		Muscedere, et al., 2015
Vollenhovia nipponica		Ohkawara and Aonuma, 2016				Ohkawara & Aonuma, 2016)		
Xylocopa appendiculata								
Agrotis ipsilon	Barrozo, et al., 2010						Barrozo, et al., 2010	
Bombyx mori			Hirashima, et al., 1999			Pophof, 2002	Pophof, 2002	
Caloptilia fraxinella							Lemmen and Evenden, 2015	
Galleria mellonella L						Abdoun, et al., 1995		
Helicoverpa zea								
Mamestra brassicae							Grosmaitre, et al., 2001	

Manduca sexta	Claassen and Kammer, 1986			Claassen and Kammer, 1986			Dolzer, et al., 2001	
Trichoplusia ni					Linn and Roelofs, 1986		Linn and Roelofs, 1986	
Gryllus bimaculatus	Adamo, et al., 1995	Rillich, et al., 2011, Rillich and Stevenson, 2011, 2015, Stevenson, et al., 2005, Stevenson and Rillich, 2017, Stevenson and Schildberger, 2013		Mizunami, et al., 2009, Unoki, et al., 2005		Abdoun, et al., 1995, Sakai, et al., 2017		
Locusta migratoria				Bacon, et al., 1995				Ma, et al., 2015
Schistocerca americana	Morris, et al., 1999, Orchard, et al., 1993, Sombati and Hoyle, 1984b			Sombati and Hoyle, 1984a				

SEROTONIN

	**Activity**	**Aggression**	**Development**	**Integration**	**Nutrition**	**Reproduction**	**Sensorimotor function**	**Social function**
Hodotermopsis sjostedti								
Leucophaea maderae	Page, 1987				Cohen, 2001			
Periplaneta americana	Goldstein and Camhi, 1991			Goldstein and Camhi, 1991			Goldstein and Camhi, 1991	
Reticulitermes flavipes					Nuss, et al., 2010			
Zootermopsis nevadensis								
Nicrophorus orbicollis						Panaitof, et al., 2016		Panaitof, et al., 2016
Tribolium castaneum	Nishi, et al., 2010							
Tribolium freemani								
Aedes albopictus								
Anopheles gambiae	Fuchs, et al., 2014							
Calliphora erythrocephala			Nässel and Laxmyr, 1983					
Drosophila melanogaster	Neckameyer, et al., 2007, Yellman, et al., 1997, Yuan, et al.,	Alekseyenko, et al., 2014, Alekseyenko, et al., 2010,	Neckameyer and Bhatt, 2012	Sitaraman, et al., 2008, Vargas, et al., 2010	Neckameyer, et al., 2007, Vargas, et al., 2010 Neckameyer,		Neckameyer, et al., 2007	

	2006, Yuan, et al., 2005	Johnson, et al., 2009			et al., 2007			
Neobellieria bullata	Dacks, et al., 2003				Dacks, et al., 2003			
Phormia regina					Haselton, et al., 2009, Long and Murdock, 1983			
Teleopsis dalmanni		Bubak, et al., 2014, Bubak, et al., 2016						
Lygus hesperus						Brent, et al., 2016		
Pyrrhocoris apterus						Chvalova, et al., 2014		
Rhodnius prolixus					Orchard, 2006			
Myzus persicae					Kaufmann, et al., 2004			
Acromyrmex echinatior								Smith, et al., 2013
Apis mellifera			Harris and Woodring, 1992	Blenau and Erber, 1998, Chen, et al., 2008, Mercer and Menzel, 1982	French, et al., 2014		Mercer and Menzel, 1982	
Bombus terrestris						Bloch, et al., 2000		
Camponotus fellah					Boulay, et al., 2000			Boulay, et al., 2000
Camponotus mus					Falibene, et al., 2012			
Diacamma sp.								
Formica japonica		Aonuma and Watanabe, 2012b	Aonuma and Watanabe, 2012b			Aonuma and Watanabe, 2012a		
Formica polyctena		Szczuka, et al., 2013						
Formica rufa		Kostowski, et al., 1975						
Harpegnathos saltator		Hoyer, et al., 2005, Penick, et al., 2014	Penick, et al., 2014			Hoyer, et al., 2005, Penick, et al., 2014		Penick, et al., 2014
Pheidole dentata	Giraldo, et al., 2016b		Giraldo, et al., 2016a, Seid and Traniello, 2005	Giraldo, et al., 2016b			Giraldo, et al., 2016b, Muscedere, et al., 2012	Giraldo, et al., 2016b, Muscedere, et al., 2012, Seid and Traniello, 2005; Sandridge et al., unpublished data
Pheidole morrisi								(Sandridge et al., unpublished data)
Polistes chinensis						Sasaki, et al., 2009		

Oecophylla smaragdina		Kamhi, et al., 2015						
Polyrhachis moesta								
Solenopsis invicta						Boulay, et al., 2001		
Streblognathus peetersi			Cuvillier-Hot and Lenoir, 2006			Cuvillier-Hot and Lenoir, 2006		Cuvillier-Hot and Lenoir, 2006
Veromessor pergandei		Muscedere, et al., 2015						
Vollenhovia nipponica		Ohkawara and Aonuma, 2016				Ohkawara and Aonuma, 2016		
Xylocopa appendiculata								
Agrotis ipsilon	Barrozo, et al., 2010					Barrozo, et al., 2010	Barrozo, et al., 2010	
Bombyx mori							Hill, et al., 2003	
Caloptilia fraxinella							Lemmen and Evenden, 2016	
Galleria mellonella L								
Helicoverpa zea					Cohen, et al., 1988			
Mamestra brassicae							Grosmaitre, et al., 2001	
Manduca sexta	Claassen and Kammer, 1986			Claassen and Kammer, 1986		Kloppenburg, et al., 1999	Dolzer, et al., 2001, Kloppenburg, et al., 1999, Kloppenburg and Mercer, 2008	
Trichoplusia ni	Linn and Roelofs, 1986					Linn and Roelofs, 1986	Linn and Roelofs, 1986	
Gryllus bimaculatus	Dyakonova, et al., 1999			Dyakonova, et al., 1999			Dyakonova, et al., 1999	
Locusta migratoria					Kaufmann, et al., 2004			
Schistocerca americana								
Schistocerca gregaria					Baines and Tyrer, 1989			Anstey, et al., 2009

DOPAMINE

	**Activity**	**Aggression**	**Development**	**Integration**	**Nutrition**	**Reproduction**	**Sensorimotor function**	**Social function**

Hodotermopsis sjostedti								
Leucophaea maderae	Page, 1987				Allen, et al., 2011			
Periplaneta americana	Weisel-Eichler and Libersat, 2002			Goldstein and Camhi, 1991			Goldstein and Camhi, 1991, Weisel-Eichler and Libersat, 2002	
Reticulitermes flavipes								
Zootermopsis nevadensis			Yaguchi, et al., 2016					Yaguchi, et al., 2016
Nicrophorus orbicollis								Panaitof, et al., 2016
Tribolium castaneum	Miyatake, et al., 2008, Nishi, et al., 2010							
Tribolium freemani								
Aedes albopictus					Fukumitsu, et al., 2012	Fukumitsu et al., 2012)		
Anopheles gambiae	Fuchs, et al., 2014							
Calliphora erythrocephala			Nässel and Laxmyr, 1983					
Drosophila melanogaster	Alekseyenko, et al., 2010, Draper, et al., 2007, Yellman, et al., 1997	Alekseyenko, et al., 2013	Neckameyer and Bhatt, 2012	Aso, et al., 2010, Claridge-Chang, et al., 2009, Cohn, et al., 2015, Kim, et al., 2007, Liu, et al., 2012, Schwaerzel, et al., 2003, Selcho, et al., 2009, Van Swinderen and Andretic, 2011, Zhang, et al., 2007		Neckameyer, 1998a, b	Van Swinderen and Andretic, 2011	
Neobellieria bullata								
Phormia regina					Long and Murdock, 1983			
Teleopsis dalmanni								
Lygus hesperus						Brent, et al., 2016		
Pyrrhocoris apterus						Chvalova, et al., 2014		
Rhodnius prolixus								
Myzus persicae								
Acromyrmex echinatior								Smith, et al., 2013
Apis mellifera	Beggs, et al., 2007, Bozic and Woodring, 1998		Harano, et al., 2007, Harris and Woodring, 1992	Blenau and Erber, 1998, Chen, et al., 2008, Mercer and	Scheiner, et al., 2002	Harano, et al., 2008, Harris and Woodring, 1995	Mercer and Menzel, 1982, Scheiner, et al., 2002	Bozic and Woodring, 1998, Wagener-Hulme, et al., 1999

				Menzel, 1982				
Bombus terrestris				Perry, et al., 2016		Bloch, et al., 2000		
Camponotus fellah								
Camponotus mus								
Diacamma sp.		Okada, et al., 2015, Shimoji, et al., 2017				Okada, et al., 2015		Okada, et al., 2015
Formica japonica		Aonuma and Watanabe, 2012b, Wada-Katsumata, et al., 2011	Aonuma and Watanabe, 2012a		Wada-Katsumata, et al., 2011	Aonuma and Watanabe, 2012a		Wada-Katsumata, et al., 2011
Formica polyctena		Szczuka, et al., 2013	Wnuk, et al., 2010					Wnuk, et al., 2010
Formica rufa								
Harpegnathos saltator		Hoyer, et al., 2005, Penick, et al., 2014	Penick, et al., 2014			Hoyer, et al., 2005, Penick, et al., 2014		Penick, et al., 2014
Pheidole dentata			Seid and Traniello, 2005					Giraldo, et al., 2016b, Muscedere, et al., 2013, Seid and Traniello, 2005; Sandridge et al., unpublished
Pheidole morrisi								(Sandridge et al., unpublished)
Polistes chinensis						Sasaki and Harano, 2007		
Oecophylla smaragdina		Kamhi, et al., 2015						
Polyrhachis moesta								
Solenopsis invicta		Vander Meer, et al., 2008				Boulay, et al., 2001	Vander Meer, et al., 2008	
Streblognathus peetersi			Cuvillier-Hot and Lenoir, 2006			Cuvillier-Hot and Lenoir, 2006		Cuvillier-Hot and Lenoir, 2006
Veromessor pergandei						Muscedere, et al., 2015		Muscedere, et al., 2015
Vollenhovia nipponica		Ohkawara and Aonuma, 2016				Ohkawara & Aonuma, 2016)		
Xylocopa appendiculata	Sasaki and Nagao, 2013							
Agrotis ipsilon								
Bombyx mori			Hirashima, et al., 1999, Noguchi and Hayakawa, 2001					
Caloptilia fraxinella							Lemmen and Evenden, 2016	

Galleria mellonella L								
Helicoverpa zea								
Mamestra brassicae								
Manduca sexta	Claassen and Kammer, 1986			Claassen and Kammer, 1986				
Trichoplusia ni								
Gryllus bimaculatus		Rillich and Stevenson, 2014		Mizunami, et al., 2009, Unoki, et al., 2005				
Locusta migratoria								
Schistocerca americana								
Schistocerca gregaria					Baines and Tyrer, 1989			

Table S2: References to studies documenting biogenic amine functions for behaviors in eight categories.

References

Abdoun, K., Mesniersabin, M., Baudrypartiaoglou, N., Nicolas, P. and Cohen, P. (1995). Separation of oviposition-stimulating peptides and myotropic factors from head extracts of *Galleria mellonella* L - Comparative effects of myotropic and non-myotropic factors on egg-laying. J. Comp. Physiol. B Biochem. Syst. Environ. Physiol. 165. 102-109.

Adamo, S. A., Linn, C. E. and Hoy, R. R. (1995). The role of neurohormonal octopamine during fight or flight behavior in the field cricket *Gryllus bimaculatus*. J. Exp. Biol. 198. 1691-1700.

Alekseyenko, O. V., Chan, Y. B., Fernandez Mde, L., Bulow, T., Pankratz, M. J. and Kravitz, E. A. (2014). Single serotonergic neurons that modulate aggression in *Drosophila*. Curr. Biol. 24. 2700-7.

Alekseyenko, O. V., Chan, Y. B., Li, R. and Kravitz, E. A. (2013). Single dopaminergic neurons that modulate aggression in *Drosophila*. Proc. Natl. Acad. Sci. U. S. A. 110. 6151-6156.

Alekseyenko, O. V., Lee, C. and Kravitz, E. A. (2010). Targeted manipulation of serotonergic neurotransmission affects the escalation of aggression in adult male *Drosophila melanogaster*. PLoS One 5. e10806.

Allen, J. M., Van Kummer, B. H. and Cohen, R. W. (2011). Dopamine as an anorectic neuromodulator in the cockroach *Rhyparobia maderae*. J. Exp. Biol. 214. 3843-9.

Anstey, M. L., Rogers, S. M., Ott, S. R., Burrows, M. and Simpson, S. J. (2009). Serotonin mediates behavioral gregarization underlying swarm formation in desert locusts. Science 323. 627-630.

Aonuma, H. and Watanabe, T. (2012a). Changes in the content of brain biogenic amine associated with early colony establishment in the queen of the ant, *Formica japonica*. PloS One 7.

Aonuma, H. and Watanabe, T. (2012b). Octopaminergic system in the brain controls aggressive motivation in the ant, *Formica japonica*. Acta Biol. Hung. 63. 63-68.

Aso, Y., Siwanowicz, I., Bracker, L., Ito, K., Kitamoto, T. and Tanimoto, H. (2010). Specific dopaminergic neurons for the formation of labile aversive memory. Curr. Biol. 20. 1445-1451.

Bacon, J. P., Thompson, K. S. J. and Stern, M. (1995). Identified octopaminergic neurons provide an arousal mechanism in the locust brain. J. Neurophysiol. 74. 2739-2743.

Baines, R. A. and Tyrer, N. M. (1989). The innervation of salivary glands. II. Physiology of excitation and modulation. J. Comp. Physiol. A 165. 407-413.

Barron, A. B., Maleszka, R., Vander Meer, R. K. and Robinson, G. E. (2007). Octopamine modulates honey bee dance behavior. Proc. Natl. Acad. Sci. U. S. A. 104. 1703-7.

Barrozo, R. B., Jarriault, D., Simeone, X., Gaertner, C., Gadenne, C. and Anton, S. (2010). Mating-induced transient inhibition of responses to sex pheromone in a male moth is not mediated by octopamine or serotonin. J. Exp. Biol. 213. 1100-6.

Beggs, K. T., Glendining, K. A., Marechal, N. M., Vergoz, V., Nakamura, I., Slesoor, K. N., et al. (2007). Queen pheromone modulates brain dopamine function in worker honey bees. Proc. Natl. Acad. Sci. U. S. A. 104. 2460-2464.

Blenau, W. and Erber, J. (1998). Behavioural pharmacology of dopamine, serotonin and putative aminergic ligands in the mushroom bodies of the honeybee (*Apis mellifera*). Behav. Brain. Res. 96. 115-124.

Bloch, G., Simon, T., Robinson, G. E. and Hefetz, A. (2000). Brain biogenic amines and reproductive dominance in bumble bees (*Bombus terrestris*). J. Comp. Physiol. A 186. 261-8.

Boulay, R., Hooper-Bui, L. M. and Woodring, J. (2001). Oviposition and oogenesis in virgin fire ant females *Solenopsis invicta* are associated with a high level of dopamine in the brain. Physiol. Entomol. 26. 294-299.

Boulay, R., Soroker, V., Godzinska, E. J., Hefetz, A. and Lenoir, A. (2000). Octopamine reverses the isolation-induced increase in trophallaxis in the carpenter ant *Camponotus fellah*. J. Exp. Biol. 203. 513-520.

Bozic, J. and Woodring, J. (1998). Variations of brain biogenic amines in mature honeybees and induction of recruitment behavior. Comp. Biochem. Physiol. Part A Mol. Integr. Physiol. 120. 737-744.

Braun, G. and Bicker, G. (1992). Habituation of an appetitive reflex in the honeybee. J. Neurophysiol. 67. 588-98.

Brent, C. S., Miyasaki, K., Vuong, C., Miranda, B., Steele, B., Brent, K. G., et al. (2016). Regulatory roles of biogenic amines and juvenile hormone in the reproductive behavior of the western tarnished plant bug (*Lygus hesperus*). J. Comp. Physiol. B Biochem. Syst. Environ. Physiol. 186. 169-79.

Bubak, A. N., Renner, K. J. and Swallow, J. G. (2014). Heightened serotonin influences contest outcome and enhances expression of high-intensity aggressive behaviors. Behav. Brain. Res. 259. 137-42.

Bubak, A. N., Yaeger, J. D., Renner, K. J., Swallow, J. G. and Greene, M. J. (2016). Neuromodulation of nestmate recognition decisions by pavement ants. PLoS One 11. e0166417.

Burke, C. J., Huetteroth, W., Owald, D., Perisse, E., Krashes, M. J., Das, G., et al. (2012). Layered reward signalling through octopamine and dopamine in *Drosophila*. Nature 492. 433-+.

Burrell, B. D. and Smith, B. H. (1995). Modulation of the honey bee (*Apis mellifera*) sting response by octopamine. J. Insect Physiol. 41. 671-680.

Certel, S. J., Leung, A., Lin, C. Y., Perez, P., Chiang, A. S. and Kravitz, E. A. (2010). Octopamine neuromodulatory effects on a social behavior decision-making network in *Drosophila* males. PLoS One 5. e13248.

Chen, Y. L., Hung, Y. S. and Yang, E. C. (2008). Biogenic amine levels change in the brains of stressed honeybees. Arch. Insect Biochem. Physiol. 68. 241-250.

Chvalova, D., Zdechovanova, L., Vaneckova, H. and Hodkova, M. (2014). Brain norepinephrine identified by mass spectrometry is associated with reproductive status of females of the linden bug *Pyrrhocoris apterus*. Comp. Biochem. Phys. B 168. 70-5.

Claassen, D. E. and Kammer, A. E. (1986). Effects of octopamine, dopamine, and serotonin on production of flight motor output by thoracic ganglia of *Manduca sexta*. J. Neurobiol. 17. 1-14.

Claridge-Chang, A., Roorda, R. D., Vrontou, E., Sjulson, L., Li, H., Hirsh, J., et al. (2009). Writing memories with light-addressable reinforcement circuitry. Cell 139. 405-15.

Cohen, R. W. (2001). Diet balancing in the cockroach *Rhyparobia madera*: Does serotonin regulate this behavior? J. Insect Behav. 14. 99-111.

Cohen, R. W., Friedman, S. and Waldbauer, G. P. (1988). Physiological control of nutrient self-selection in *Heliothis zea* larvae: the role of serotonin. J. Insect Physiol. 34. 935-940.

Cohen, R. W., Mahoney, D. A. and Can, H. D. (2002). Possible regulation of feeding behavior in cockroach nymphs by the neurotransmitter octopamine. J. Insect Behav. 15. 37-50.

Cohn, R., Morantte, I. and Ruta, V. (2015). Coordinated and compartmentalized neuromodulation shapes sensory processing in *Drosophila*. Cell 163. 1742-1755.

Cuvillier-Hot, V. and Lenoir, A. (2006). Biogenic amine levels, reproduction and social dominance in the queenless ant *Streblognathus peetersi*. Naturwissenschaften 93. 149-153.

Dacks, A. M., Nickel, T. and Mitchell, B. K. (2003). An examination of serotonin and feeding in the flesh fly *Neobellieria bullata* (Sarcophagidae : Diptera). J. Insect Behav. 16. 1-21.

Djernaes, M., Klass, K. D., Picker, M. D. and Damgaard, J. (2011). Phylogeny of cockroaches (Insecta, Dictyoptera, Blattodea), with placement of aberrant taxa and exploration of out-group sampling. Syst. Entomol. 37. 65-83.

Dolzer, J., Krannich, S., Fischer, K. and Stengl, M. (2001). Oscillations of the transepithelial potential of moth olfactory sensilla are influenced by octopamine and serotonin. J. Exp. Biol. 204. 2781-2794.

Draper, I., Kurshan, P. T., McBride, E., Jackson, F. R. and Kopin, A. S. (2007). Locomotor activity is regulated by D2-like receptors in *Drosophila*: an anatomic and functional analysis. Dev. Neurobiol. 67. 378-93.

Dyakonova, V. E., Schurmann, F. and Sakharov, D. A. (1999). Effects of serotonergic and opioidergic drugs on escape behaviors and social status of male crickets. Naturwissenschaften 86. 435-7.

Falibene, A., Rössler, W. and Josens, R. (2012). Serotonin depresses feeding behaviour in ants. J. Insect Physiol. 58. 7-17.

French, A. S., Simcock, K. L., Rolke, D., Gartside, S. E., Blenau, W. and Wright, G. A. (2014). The role of serotonin in feeding and gut contractions in the honeybee. J. Insect Physiol. 61. 8-15.

Fuchs, S., Rende, E., Crisanti, A. and Nolan, T. (2014). Disruption of aminergic signalling reveals novel compounds with distinct inhibitory effects on mosquito reproduction, locomotor function and survival. Sci. Rep. 4. 5526.

Fukumitsu, Y., Irie, K., Satho, T., Aonuma, H., Dieng, H., Ahmad, A. H., et al. (2012). Elevation of dopamine level reduces host-seeking activity in the adult female mosquito *Aedes albopictus*. Parasite Vector 5.

Giraldo, Y. M., Kamhi, J. F., Fourcassie, V., Moreau, M., Robson, S. K., Rusakov, A., et al. (2016a). Lifespan behavioural and neural resilience in a social insect. P. Roy. Soc. B-Biol. Sci. 283.

Giraldo, Y. M., Rusakov, A., Diloreto, A., Kordek, A. and Traniello, J. F. (2016b). Age, worksite location, neuromodulators, and task performance in the ant *Pheidole dentata*. Behav. Ecol. Sociobiol. 70. 1441-1455.

Goldstein, R. S. and Camhi, J. M. (1991). Different effects of the biogenic amines dopamine, serotonin and octopamine on the thoracic and abdominal portions of the escape circuit in the cockroach. J. Comp. Physiol. A 168. 103-112.

Grosmaitre, X., Marion-Poll, F. and Renou, M. (2001). Biogenic amines modulate olfactory receptor neurons firing activity in *Mamestra brassicae*. Chem Senses 26. 653-61.

Hammer, M. and Menzel, R. (1995). Learning and memory in the honeybee. J. Neurosci. 15. 1617-1630.

Harano, K., Sasaki, K., Nagao, T. and Sasaki, M. (2008). Influence of age and juvenile hormone on brain dopamine level in male honeybee (*Apis mellifera*): association with reproductive maturation. J. Insect Physiol. 54. 848-53.

Harano, K., Sasaki, M. and Sasaki, K. (2007). Effects of reproductive state on rhythmicity, locomotor activity and body weight in the European honeybee, Apis mellifera queens (Hymenoptera, Apini). Sociobiology 50. 189-200.

Harris, J. W. and Woodring, J. (1992). Effects of stress, age, season, and source colony on levels of octopamine, dopamine and serotonin in the honey bee (*Apis mellifera* L.) brain. J. Insect Physiol. 38. 29.

Harris, J. W. and Woodring, J. (1995). Elevated brain dopamine levels associated with ovary development in queenless worker honey bees (*Apis mellifera* L.). Comp. Biochem. Physiol. 3. 271-279.

Haselton, A. T., Downer, K. E., Zylstra, J. and Stoffolano, J. G. (2009). Serotonin inhibits protein feeding in the blow fly, *Phormia regina* (Meigen). J. Insect Behav. 22. 452-463.

Hill, E. S., Okada, K. and Kanzaki, R. (2003). Visualization of modulatory effects of serotonin in the silkmoth antennal lobe. J. Exp. Biol. 206. 345-352.

Hirashima, A., Suetsugu, E., Hirokado, S., Kuwano, E., Taniguchi, E. and Eto, M. (1999). Effect of octopamine on the activity of juvenile-hormone esterase in the silkworm *Bombyx mori* and the red flour beetle *Tribolium freemani*. Gen. Comp. Endocrinol. 116. 373-81.

Hoyer, S. C., Eckart, A., Herrel, A., Zars, T., Fischer, S. A., Hardie, S. L., et al. (2008). Octopamine in male aggression of *Drosophila*. Curr. Biol. 18. 159-167.

Hoyer, S. C., Liebig, Liebig, J. and Rössler, W. (2005). Biogenic amines in the ponerine ant *Harpegnathos saltator*: serotonin and dopamine immunoreactivity in the brain. Arthropod Struct. Dev. 34. 429-440.

Ishikawa, Y., Aonuma, H., Sasaki, K. and Miura, T. (2016). Tyraminergic and octopaminergic modulation of defensive behavior in termite soldier. PLoS One 11. e0154230.

Johnson, O., Becnel, J. and Nichols, C. D. (2009). Serotonin 5-HT(2) and 5-HT(1A)-like receptors differentially modulate aggressive behaviors in *Drosophila melanogaster*. Neuroscience 158. 1292-300.

Kamhi, J. F., Nunn, K., Robson, S. K. A. and Traniello, J. F. A. (2015). Polymorphism and division of labour in a socially complex ant: neuromodulation of aggression in the Australian weaver ant, *Oecophylla smaragdina*. P. Roy. Soc. B-Biol. Sci. 282.

Kaufmann, L., Schurmann, F., Yiallouros, M., Harrewijn, P. and Kayser, H. (2004). The serotonergic system is involved in feeding inhibition by pymetrozine. Comparative studies on a locust (*Locusta migratoria*) and an aphid (*Myzus persicae)*. Comparative Biochemical and Physiology C Toxicology & Pharmacology 138. 469-83.

Kim, Y. C., Lee, H. G. and Han, K. A. (2007). D1 dopamine receptor dDA1 is required in the mushroom body neurons for aversive and appetitive learning in *Drosophila*. J. Neurosci. 27. 7640-7.

Kloppenburg, P. and Erber, J. (1995). The modulatory effects of serotonin and octopamine in the visual system of the honey bee (*Apis mellifera* L) .2. Electrophysiological analysis of motion sensitive neurons in the lobula. J. Comp. Physiol. A 176. 119-129.

Kloppenburg, P., Ferns, D. and Mercer, A. R. (1999). Serotonin enhances central olfactory neuron responses to female sex pheromone in the male sphinx moth *Manduca sexta*. J. Neurosci. 19. 8172-81.

Kloppenburg, P. and Mercer, A. R. (2008). Serotonin modulation of moth central olfactory neurons. Annu. Rev. Entomol. 53. 179-90.

Kostowski, W., Tarchalska-Krynska, B. and Markowska, L. (1975). Aggressive behavior and brain serotonin and catecholamines in ants (*Formica rufa*). Pharmacol. Biochem. Behav. 3. 717-9.

Koyama, S., Matsui, S., Satoh, T. and Sasaki, K. (2015). Octopamine and cooperation: octopamine regulates the disappearance of cooperative behaviours between genetically unrelated founding queens in the ant. Biol. Lett. 11. 20150206.

Lee, H. G., Seong, C. S., Kim, Y. C., Davis, R. L. and Han, K. A. (2003). Octopamine receptor OAMB is required for ovulation in *Drosophila melanogaster*. Dev. Biol. 264. 179-90.

Lemmen, J. and Evenden, M. (2015). Environmental conditions terminate reproductive diapause and influence pheromone perception in the long-lived moth *Caloptilia fraxinella*. Physiol. Entomol. 40. 30-42.

Lemmen, J. K. and Evenden, M. L. (2016). The roles of juvenile hormone and biogenic amines on pheromone response plasticity and diapause termination in male *Caloptilia fraxinella*. Entomol. Exp. Appl. 158. 184-201.

Linn, C. E. and Roelofs, W. L. (1986). Modulatory effects of octopamine and serotonin on male sensitivity and periodicity of response to sex pheromone in the cabbage looper moth, *Trichoplusia ni*. Arch. Insect Biochem. Physiol. 3. 161-171.

Liu, C., Placais, P. Y., Yamagata, N., Pfeiffer, B. D., Aso, Y., Friedrich, A. B., et al. (2012). A subset of dopamine neurons signals reward for odour memory in *Drosophila*. Nature 488. 512-6.

Long, T. F. and Murdock, L. L. (1983). Stimulation of blowfly feeding behavior by octopaminergic drugs. Proc. Natl. Acad. Sci. U. S. A. 80. 4159-4163.

Luo, J. N., Lushchak, O. V., Goergen, P., Williams, M. J. and Nassel, D. R. (2014). *Drosophila* insulin-producing cells are differentially modulated by serotonin and octopamine receptors and affect cocial behavior. PloS One 9.

Ma, Z., Guo, X., Lei, H., Li, T., Hao, S. and Kang, L. (2015). Octopamine and tyramine respectively regulate attractive and repulsive behavior in locust phase changes. Sci. Rep. 5. 8036.

Menzel, R., Heyne, A., Kinzel, C., Gerber, B. and Fiala, A. (1999). Pharmacological dissociation between the reinforcing, sensitizing, and response-releasing functions of reward in honeybee classical conditioning. Behav. Neurosci. 113. 744-754.

Mercer, A. R. and Menzel, R. (1982). The effects of biogenic amines on conditioned and unconditioned responses to olfactory stimuli in the honeybee *Apis mellifera*. J. Comp. Physiol. 145. 363-368.

Misof, B., Liu, S. L., Meusemann, K., Peters, R. S., Donath, A., Mayer, C., et al. (2014). Phylogenomics resolves the timing and pattern of insect evolution. Science 346. 763-767.

Miyatake, T., Tabuchi, K., Sasaki, K., Okada, K., Katayama, K. and Moriya, S. (2008). Pleiotropic antipredator strategies, fleeing and feigning death, correlated with dopamine levels in *Tribolium castaneum*. Anim. Behav. 75. 113-121.

Mizunami, M., Unoki, S., Mori, Y., Hirashima, D., Hatano, A. and Matsumoto, Y. (2009). Roles of octopaminergic and dopaminergic neurons in appetitive and aversive memory recall in an insect. BMC Biol. 7.

Moreau, C. S. and Bell, C. D. (2013). Testing the museum versus cradle tropical biological diversity hypothesis: phylogeny, diversification, and ancestral biogeographic range evolution of the ants. Evolution doi: 10.1111/evo.12105.

Morris, O. T., Duch, C. and Stevenson, P. A. (1999). Differential activation of octopaminergic (DUM) neurones via proprioceptors responding to flight muscle contractions in the locust. J. Exp. Biol. 202. 3555-3564.

Müller, U. (1997). Neuronal cAMP-dependent protein kinase type II is concentrated in mushroom bodies of *Drosophila melanogaster* and the honeybee *Apis mellifera*. J. Neurobiol. 33. 33-44.

Muscedere, M. L., Djermoun, A. and Traniello, J. F. A. (2013). Brood-care experience, nursing performance, and neural development in the ant *Pheidole dentata*. Behav. Ecol. Sociobiol. 67. 775-784.

Muscedere, M. L., Helms Cahan, S., Helms, K. R. and Traniello, J. F. (2015). Geographic and life-history variation in ant queen colony founding correlates with brain amine levels. Behav. Ecol. 27. 271-278.

Muscedere, M. L., Johnson, N., Gillis, B. C., Kamhi, J. F. and Traniello, J. F. A. (2012). Serotonin modulates worker responsiveness to trail pheromone in the ant *Pheidole dentata*. J. Comp. Physiol. A 198. 219-227.

Nässel, D. R. and Laxmyr, L. (1983). Quantitative determination of biogenic amines and DOPA in the CNS of adult and larval blowflies, *Calliphora erythrocephala*. Comp. Biochem. Phys. C 75. 259-265.

Neckameyer, W. S. (1998a). Dopamine and mushroom bodies in Drosophila: Experience-dependent and -independent aspects of sexual behavior. Learn. Mem. 5. 157-165.

Neckameyer, W. S. (1998b). Dopamine modulates female sexual receptivity in *Drosophila melanogaster*. J. Neurogenet. 12. 101-114.

Neckameyer, W. S. and Bhatt, P. (2012). Neurotrophic actions of dopamine on the development of a serotonergic feeding circuit in *Drosophila melanogaster*. BMC Neurosci. 13. 26.

Neckameyer, W. S., Coleman, C. M., Eadie, S. and Goodwin, S. F. (2007). Compartmentalization of neuronal and peripheral serotonin synthesis in *Drosophila melanogaster*. Genes Brain Behav. 6. 756-69.

Nishi, Y., Sasaki, K. and Miyatake, T. (2010). Biogenic amines, caffeine and tonic immobility in *Tribolium castaneum*. J. Insect Physiol. 56. 622-628.

Noguchi, H. and Hayakawa, Y. (2001). Dopamine is a key factor for the induction of egg diapause of the silkworm, *Bombyx mori*. Eur. J. Biochem. 268. 774-80.

Nuss, A. B., Forschler, B. T., Crim, J. W., TeBrugge, V., Pohl, J. and Brown, M. R. (2010). Molecular characterization of neuropeptide F from the eastern subterranean termite *Reticulitermes flavipes* (Kollar) (Isoptera: Rhinotermitidae). Peptides 31. 419-428.

Ohkawara, K. and Aonuma, H. (2016). Changes in the levels of biogenic amines associated with aggressive behavior of queen in the social parasite ant *Vollenhovia nipponica*. Insectes Soc. 63. 257-264.

Okada, Y., Sasaki, K., Miyazaki, S., Shimoji, H., Tsuji, K. and Miura, T. (2015). Social dominance and reproductive differentiation mediated by dopaminergic signaling in a queenless ant. J. Exp. Biol. 218. 1091-8.

Orchard, I. (2006). Serotonin: a coordinator of feeding-related physiological events in the blood-gorging bug, *Rhodnius prolixus*. Comp. Biochem. Physiol. A Mol. Integr. Physiol. 144. 316-24.

Orchard, I., Ramirez, J. M. and Lange, A. B. (1993). A multifunctional role for octopamine in locust flight. Annu. Rev. Entomol. 38. 227-249.

Page, T. L. (1987). Serotonin phase-shifts the circadian rhythm of locomotor activity in the cockroach. J. Biol. Rhythms 2. 23-34.

Panaitof, S. C., Yaeger, J. D. W., Speer, J. P. and Renner, K. J. (2016). Biparental behavior in the burying beetle *Nicrophorus orbicollis*: a role for dopamine? Curr. Zool. 62. 285-291.

Penick, C. A., Brent, C. S., Dolezal, K. and Liebig, J. (2014). Neurohormonal changes associated with ritualized combat and the formation of a reproductive hierarchy in the ant *Harpegnathos saltator*. J. Exp. Biol. 217. 1496-503.

Perry, C. J., Baciadonna, L. and Chittka, L. (2016). Unexpected rewards induce dopamine-dependent positive emotion-like state changes in bumblebees. Science 353. 1529-1531.

Pophof, B. (2002). Octopamine enhances moth olfactory responses to pheromones, but not those to general odorants. J. Comp. Physiol. A 188. 659-662.

Regier, J. C., Mitter, C., Zwick, A., Bazinet, A. L., Cummings, M. P., Kawahara, A. Y., et al. (2013). A large-scale, higher-level, molecular phylogenetic study of the insect order Lepidoptera (moths and butterflies). PloS One 8.

Rillich, J., Schildberger, K. and Stevenson, P. A. (2011). Octopamine and occupancy: an aminergic mechanism for intruder-resident aggression in crickets. P. Roy. Soc. B-Biol. Sci. 278. 1873-1880.

Rillich, J. and Stevenson, P. A. (2011). Winning fights induces hyperaggression via the action of the biogenic amine octopamine in crickets. PLoS One 6.

Rillich, J. and Stevenson, P. A. (2014). A fighter's comeback: dopamine is necessary for recovery of aggression after social defeat in crickets. Horm. Behav. 66. 696-704.

Rillich, J. and Stevenson, P. A. (2015). Releasing stimuli and aggression in crickets: octopamine promotes escalation and maintenance but not initiation. Front. Behav. Neurosci. 9. 95.

Robinson, G. E., Heuser, L. M., Le Conte, Y., Lenquette, F. and Hollingworth, R. M. (1999). Neurochemicals aid bee nestmate recognition. Nature 399. 534-535.

Sadaf, S., Reddy, O. V., Sane, S. P. and Hasan, G. (2015). Neural control of wing coordination in flies. Curr. Biol. 25. 80-6.

Sakai, M., Kumashiro, M., Matsumoto, Y., Ureshi, M. and Otsubo, T. (2017). Reproductive behavior and physiology in the cricket *Gryllus bimaculatus*. in: Horch, H. W., Mito, T., Popadic, A., Ohuchi, H. and Noji, S., (Eds.), The cricket as a model organism: Development, regeneration, and behavior, Springer Verlag, Japan, 2017, pp. 245-269.

Saraswati, S., Fox, L. E., Soll, D. R. and Wu, C. F. (2004). Tyramine and octopamine have opposite effects on the locomotion of *Drosophila* larvae. J. Neurobiol. 58. 425-41.

Sasaki, K. and Harano, K. I. (2007). Potential effects of tyramine on the transition to reproductive workers in honeybees (*Apis mellifera* L.). Physiol. Entomol. 32. 194-198.
Sasaki, K. and Nagao, T. (2013). Juvenile hormone-dopamine systems for the promotion of flight activity in males of the large carpenter bee *Xylocopa appendiculata*. Naturwissenschaften 100. 1183-6.
Sasaki, K., Yamasaki, K., Tsuchida, K. and Nagao, T. (2009). Gonadotropic effects of dopamine in isolated workers of the primitively eusocial wasp, *Polistes chinensis*. Naturwissenschaften 96. 625-9.
Scheiner, R., Pluckhahn, S., Oney, B., Blenau, W. and Erber, J. (2002). Behavioural pharmacology of octopamine, tyramine and dopamine in honey bees. Behav. Brain. Res. 136. 545-53.
Schmidt, C. (2013). Molecular phylogenetics of ponerine ants (Hymenoptera: Formicidae: Ponerinae). Zootaxa 3647. 201-250.
Schulz, D. J., Barron, A. B. and Robinson, G. E. (2002a). A role for octopamine in honey bee division of labor. Brain Behav. Evol. 60. 350-359.
Schulz, D. J. and Robinson, G. E. (2001). Octopamine influences division of labor in honey bee colonies. J. Comp. Physiol. A 187. 53-61.
Schulz, D. J., Sullivan, J. P. and Robinson, G. E. (2002b). Juvenile hormone and octopamine in the regulation of division of labor in honey bee colonies. Horm. Behav. 42. 222-231.
Schwaerzel, M., Monastirioti, M., Scholz, H., Friggi-Grelin, F., Birman, S. and Heisenberg, M. (2003). Dopamine and octopamine differentiate between aversive and appetitive olfactory memories in *Drosophila*. J. Neurosci. 23. 10495-502.
Seid, M. A. and Traniello, J. F. A. (2005). Age-related changes in biogenic amines in individual brains of the ant *Pheidole dentata*. Naturwissenschaften 92. 198-201.
Selcho, M., Pauls, D., Han, K. A., Stocker, R. F. and Thum, A. S. (2009). The role of dopamine in *Drosophila* larval classical olfactory conditioning. PLoS One 4. e5897.
Shimoji, H., Aonuma, H., Miura, T. and Tsuji, K. (2017). Queen contact and among-worker interactions dually suppress worker brain dopamine as a potential regulator of reproduction in an ant. Behav. Ecol. Sociobiol. 71. 35.
Sitaraman, D., Zars, M., Laferriere, H., Chen, Y. C., Sable-Smith, A., Kitamoto, T., et al. (2008). Serotonin is necessary for place memory in *Drosophila*. Proc. Natl. Acad. Sci. U. S. A. 105. 5579-84.
Smith, A. R., Muscedere, M. L., Seid, M. A., Traniello, J. F. and Hughes, W. O. (2013). Biogenic amines are associated with worker task but not patriline in the leaf-cutting ant *Acromyrmex echinatior*. J. Comp. Physiol. A 199. 1117-1127.
Sombati, S. and Hoyle, G. (1984a). Central nervous sensitization and dishabituation of reflex action in an insect by the neuromodulator octopamine. J. Neurobiol. 15. 455-480.
Sombati, S. and Hoyle, G. (1984b). Generation of specific behaviors in a locust by local release into neuropil of the natural neuromodulator octopamine. J. Neurobiol. 15. 481-506.
Song, H. J., Amedegnato, C., Cigliano, M. M., Desutter-Grandcolas, L., Heads, S. W., Huang, Y., et al. (2015). 300 million years of diversification: elucidating the patterns of orthopteran evolution based on comprehensive taxon and gene sampling. Cladistics 31. 621-651.
Song, N., Liang, A. P. and Bu, C. P. (2012). A molecular phylogeny of Hemiptera inferred from mitochondrial genome sequences. PloS One 7.
Stevenson, P. A., Dyakonova, V., Rillich, J. and Schildberger, K. (2005). Octopamine and experience-dependent modulation of aggression in crickets. J. Neurosci. 25. 1431-1441.
Stevenson, P. A. and Rillich, J. (2017). Neuromodulators and the control of aggression in crickets. in: Wilson Horch, H., Mito, T., Popadic, A., Ohuchi, H. and Noji, S., (Eds.), The cricket as a model organism: development, regeneration, and behavior, Springer, Tokyo, Japan, 2017.
Stevenson, P. A. and Schildberger, K. (2013). Mechanisms of experience dependent control of aggression in crickets. Curr. Opin. Neurobiol. 23.

Szczuka, A., Korczynska, J., Wnuk, A., Symonowicz, B., Szwacka, A. G., Mazurkiewicz, P., et al. (2013). The effects of serotonin, dopamine, octopamine, and tyramine on behavior of workers of the ant *Formica polyctena* during dyadic aggression tests. Acta Neurobiologiae Experimentalis 73. 495-520.

Unoki, S., Matsumoto, Y. and Mizunami, M. (2005). Participation of octopaminergic reward system and dopaminergic punishment system in insect olfactory learning revealed by pharmacological study. Eur. J. Neurosci. 22. 1409-1416.

Van Swinderen, B. and Andretic, R. (2011). Dopamine in *Drosophila*: setting arousal thresholds in a miniature brain. P. Roy. Soc. B-Biol. Sci. 278. 906-13.

Vander Meer, R. K., Preston, C. A. and Hefetz, A. (2008). Queen regulates biogenic amine level and nestmate recognition in workers of the fire ant, *Solenopsis invicta*. Naturwissenschaften 95. 1155-1158.

Vargas, M. A., Luo, N., Yamaguchi, A. and Kapahi, P. (2010). A role for S6 kinase and serotonin in postmating dietary switch and balance of nutrients in *D. melanogaster*. Curr. Biol. 20. 1006-11.

Wada-Katsumata, A., Yamaoka, R. and Aonuma, H. (2011). Social interactions influence dopamine and octopamine homeostasis in the brain of the ant *Formica japonica*. J. Exp. Biol. 214. 1707-1713.

Wagener-Hulme, C., Kuehn, J. C., Schulz, D. J. and Robinson, G. E. (1999). Biogenic amines and division of labor in honey bee colonies. J. Comp. Physiol. A 184. 471-479.

Wang, Y., Li, H., Wang, P., Song, F. and Cai, W. Z. (2014). Comparative mitogenomics of plant bugs (Hemiptera: Miridae): Identifying the AGG codon reassignments between serine and lysine. PloS One 9.

Weisel-Eichler, A. and Libersat, F. (2002). Are monoaminergic systems involved in the lethargy induced by a parasitoid wasp in the cockroach prey? J. Comp. Physiol. A 188. 315-24.

Wiegmann, B. M., Trautwein, M. D., Winkler, I. S., Barr, N. B., Kim, J. W., Lambkin, C., et al. (2011). Episodic radiations in the fly tree of life. Proc. Natl. Acad. Sci. U. S. A. 108. 5690-5695.

Wnuk, A., Wiater, M. and Godzinska, E. J. (2010). Effect of past and present behavioural specialization on brain levels of biogenic amines in workers of the red wood ant *Formica polyctena*. Physiol. Entomol.

Yaguchi, H., Inoue, T., Sasaki, K. and Maekawa, K. (2016). Dopamine regulates termite soldier differentiation through trophallactic behaviours. Royal Society Open Science 3. 150574.

Yellman, C., Tao, H., He, B. and Hirsh, J. (1997). Conserved and sexually dimorphic behavioral responses to biogenic amines in decapitated *Drosophila*. Proc. Natl. Acad. Sci. U. S. A. 94. 4131-6.

Yuan, Q., Joiner, W. J. and Sehgal, A. (2006). A sleep-promoting role for the *Drosophila* serotonin receptor 1A. Curr. Biol. 16. 1051-62.

Yuan, Q., Lin, F., Zheng, X. and Sehgal, A. (2005). Serotonin modulates circadian entrainment in Drosophila. Neuron 47. 115-27.

Zhang, K., Guo, J. Z., Peng, Y., Xi, W. and Guo, A. (2007). Dopamine-mushroom body circuit regulates saliency-based decision-making in *Drosophila*. Science 316. 1901-4.

Zhou, C., Rao, Y. and Rao, Y. (2008). A subset of octopaminergic neurons are important for *Drosophila* aggression. Nat. Neurosci. 11. 1059-1067.
